# Tact

**Published:** 1893-04

**Authors:** James Shepherd

**Affiliations:** Boston


### TACT.[1]

BY JAMES SHEPHERD, D.M.D., BOSTON.

One of the characteristics of the present generation, and perhaps particularly of the American people, is the careful, intelligent thought that is put into every undertaking, however trivial. Whether it is the purchase of a house or a camera, we try to get the best we can for the money, and are not satisfied unless it comes up to our idea of what it should be. If our watch is to be repaired, we carry it to a skilful workman rather than trust it to a charlatan, who may utterly ruin it. If we wish to know if the water we are drinking is pure, we carry a sample to an expert chemist; if a surgical operation is to be performed, the best skill is none too good. The intelligent person of to-day would no more think of going to a dentist of doubtful reputation than they would trust the examination of their eyes to an unskilled oculist.

We can see that manipulative ability is a quality the possession of which is absolutely necessary to the successful dental practitioner, and any one who does not have it will find the road before him rough indeed.

But the success of the dentist does not lie wholly in his dexterity. Manual skill is not the only quality that has brought success to prominent practitioners. That this is a very important factor none will deny, but if this were all, how account for the small patronage that falls to the lot of some able men?

For a moment let us put ourselves in the place of our patients. There are some who are so conscientious and methodical that their semiannual visits to us are made with the regularity of clock-work. There is little to be done, and as the operation is not painful, they pass their time in thoughtful meditation, indulging in a nap now and then, or joining in a pleasant chat at some opportune moment. There is, however, another and by far larger class, who look upon their visits in another light.

Patients tell us how much mental suffering they have passed through before they could really make up their minds to enter the office to make an appointment, and driven then, perhaps, by the preponderance of physical pain; of sleepless nights passed just before an operation which, in reality, was not particularly painful,

---

[1] Read before the Harvard Odontological Society, November 30, 1892.

and acknowledge that the greatest suffering was in the anticipation. Do such people desire in their dentist anything besides manipulative ability? Are they satisfied with knowing that the operation has been skilfully performed, regardless of rough treatment at the hands of the operator? They cannot expect to have a painful operation performed painlessly, but they do have a right to expect that the rough places will be made as smooth as possible. To do this something more is necessary than delicacy of touch: we must have an influence over them even before they enter the chair.

But the dispositions we have to deal with are very varied. There is the sensitive patient, with highly-developed nervous organization so prevalent at the present day; the business and professional man, cool and collected in the stock exchange or on the lawyer's stand, but who finds the perspiration start on his forehead when he thinks of an hour's sitting in the doctor's chair; there is the victim of nervous prostration; children, whose minds have been filled with many a tale of woe, and scores of others, too well known to be mentioned.

To lay down a set of iron rules, according to which patients having all these varieties of dispositions should be treated, would be impossible, and it is just here that a very important element in a dentist's make-up is called for, and is perhaps as well described by the word "tact" as any other.

To have the proper amount of sympathy, and yet not so much as to take away the courage; to be able to show the patient that it is better to relax, and not keep themselves at such a high point of tension, that the result will be prostration as soon as the strain is removed, and yet be able to make them brace up at just the right time and bear with fortitude some unusually severe shock; this, I say, requires tact, and is a quality that can scarcely be prized too highly, for with it true success is possible, without it it is beyond our reach.

If we chance to have in our chair a college athlete, who has accustomed himself to all kinds of hardship so that he may cope successfully with the enemy in the field, we do not want to waste any sympathy on him; he would not like it; a half-indifferent air would suit him better; yet we must let him know that we are alive to the fact that he is taking, without a murmur, what most mortals would cower under.

Sympathy is a good thing, and no dentist should be without it, but it must sometimes go masked.

Among the most difficult of patients to get on with are the exceedingly nervous and the professional invalid, who think others must share with them their every ache. If we sympathize with these too strongly it will take from them what little fortitude they have, but if we can distract their attention from themselves and gradually make them see that they really can endure pain, we score a point for both sides. A bit of sarcasm works well at times in getting up a little combativeness, acting as a counter-irritant; in all this, however, we must be very careful to let them see that at heart they have our sympathy. But woe to the dentist who betrays a grain of nervousness or impatience; it will be detected immediately by his patient, and the trials of each are increased fourfold.

As a rule these patients have to be entertained. By this I do not mean that it is necessary or well to be constantly indulging in story-telling, as is the habit of some. To be incessantly talking would, to me at least, be too great a tax, especially as so much of our work requires concentration of thought, but there are natural intermissions when a bit of conversation will be a relief to both parties.

There are some who amuse themselves by working out problems in geometry or by reciting to themselves poetry, but for the average patient to sit two hours in a chair with not a thought from the busy operator before him is tedious.

To be able to "manage" our patients without their knowing it is a great achievement. Many desire the worst cavities attended to first. On general principles this may be the wisest course to pursue, but not always. Some, if we should begin with the most sensitive operation, will be discouraged at the start, while if we take a more simple one we gradually allay their nervous dread, so that they quietly endure that which if attempted at first would have been borne with difficulty. Of course there are cases demanding immediate attention, and we must do the best we can, but sometimes here a temporary stopping is required.

While this is a good general rule for regular patients, it applies with greater force to new ones. One of the very first things we want to do is to get the confidence of our patrons.

If, instead of starting at once on a cavity that is difficult of access, exceedingly painful perhaps at the same time, and putting in a filling that cannot be appreciated by them (although we may have put into it all the skill we have at command), we take one that we can finish quickly, easily, and the good points be readily seen by even an unskilled eye, we have their respect, which once gained will carry them and us over the more difficult places.

There is another point where a patient's wishes are to be set aside by our better judgment, and that is in the amount they can endure at one sitting. Some think that after they once have their courage up they would prefer to spend the whole day in the chair, if there is much to be done, rather than spread it over a number of sittings. This may work well in some isolated cases, but generally it is not wise. I have in mind the case of a gentleman who went to a dentist every day for a week, spending the whole of each day in the chair. After the first two or three days, his nerves were so affected that he not only had the strain during the day, but all night long he had it in his dreams. The result was that for fifteen years he carried the dread of that week, and had nothing done to his teeth. Now, by taking his medicine in smaller doses, he does not mind it, and comes for an examination every six months or a year.

We should lay it down as an iron rule that nothing short of the best work will satisfy us, and if we are satisfied surely our patients will be. There are instances, however, when the best work for the case in hand is not the most thorough.

Frequently we have some invalid or very nervous patient who cannot endure the strain of a long operation, and in such a case we are justified in temporizing, or doing work that under ordinary circumstances we would not consider up to the standard. It is far preferable to put in a filling that will preserve the tooth, although from our æsthetic stand-point it may not be what we would prefer, rather than entirely discourage them by a tedious operation so that utter neglect of the teeth in the future will be the result.

We should answer as intelligently as possible all questions that may be asked. It is pleasant to think that our patients are interested in the work. At all times we should tell them the truth, and nothing but the truth, but occasionally it is better for them not to know the *whole* truth,—*e.g.*, when I apply a preparation of arsenious acid to destroy a pulp I do not say that I am using arsenic, for the very idea of having a deadly poison in their mouth might be anything but pleasant to some. I am particular, however, to tell them that what I am using is powerful, and they must be careful not to remove it. Just so in the use of other poisons, we can satisfy there curiosity without causing anxiety by giving them the whole truth.

Perhaps there is greater opportunity to show tact in handling children than in any other class of patients. In the first place, begin with the parents if possible. Tell the mother never to mention in the presence of her children that she has had a hard time at the dentist's. The little minds are sure to catch it up and treasure it, and they easily form the idea that it is a dreadful thing to have their teeth attended to, when, nine cases out of ten, if they come with an unprejudiced mind, they do not find it bad at all.

Generally I like to have the child alone, breaking over the rule perhaps the first time, when a simple cavity is taken or the teeth cleansed. If honesty is to be practised with adults, it surely should be with children. Never deceive. Some seem to think they accomplish much if they tell the child they intend to only look at a tooth, and then, without warning, put the forceps on and extract it. They may have succeeded well in this one case, but they never get the confidence of that child again. Once secure the confidence of the child, and the way before you is comparatively smooth. Better to keep your patience for one or two sittings and win the child over to your side, than lose it at the start and have a struggle with him ever after.

The deciduous teeth are usually not very sensitive. Of course the sensation produced by the instrument is not pleasant, but if we use a little tact we can make them almost enjoy it. The little mind that can make a baby out of a rag, or a horse out of a stick, can easily be led to think that it is great fun to have the teeth cared for.

As a rule the hand instruments are preferred to the engine, but with a little tact the latter can be used. Often the most disagreeable part of this is the noise, and of course it is impossible to keep them from noticing it, but if we tell them that it makes a great noise that is very funny, we can often make them laugh over what if presented in another way might call forth a tear. Sometimes, of course, a cavity will be sensitive, but even here we can help them: appeal to their bravery, and tell them it is a good chance to show their courage, and they respond nobly.

Let us remember that we have accomplished but half our mission when we can skilfully perform an operation, the other half is to be able to manage our patients, and that can only be done by tact.

